# Predictors of Bilateral Disease in Low-Risk Papillary Thyroid Cancer: Histopathologic Insights and Preoperative Ultrasonography

**DOI:** 10.1245/s10434-024-16352-z

**Published:** 2025-01-14

**Authors:** P. M. Rodriguez Schaap, A. Papachristos, H. Serrao-Brown, A. Aniss, E. J. M. Nieveen van Dijkum, A. J. Gill, L. Delbridge, A. F. Engelsman, S. Sidhu, M. Sywak

**Affiliations:** 1 Department of Surgery, Amsterdam University Medical Centres, Location VUmc, Cancer Center Amsterdam, Amsterdam, The Netherlands; 2 Department of Endocrine Surgery, Royal North Shore Hospital, University of Sydney, Sydney, Australia; 3 Northern Clinical School, Sydney Medical School, Faculty of Medicine and Health, University of Sydney, Sydney, Australia; 4 NSW Health Pathology, Department of Pathology, Royal North Shore Hospital, St. Leonards, Australia; 5 Endocrine Surgery Unit, University of Sydney, St. Leonards, Australia

**Keywords:** Thyroid cancer, Hemithyroidectomy, Multifocality, Bilaterality, Neck ultrasound

## Abstract

**Background:**

With the current shift toward de-escalation of surgical management in low-risk papillary thyroid cancer (PTC), understanding predictors and the clinical significance of additional tumors in the contralateral lobe is important. This study investigated the histopathologic predictors of bilateral disease in low-risk PTC patients and the utility of preoperative ultrasonography in guiding completion thyroidectomy decisions.

**Methods:**

Patients treated with total thyroidectomy (TT) for low-risk PTCs (< 4 cm) at the Endocrine Surgical Unit of the Royal North Shore Hospital, University of Sydney from 2013 to 2020 were identified from a prospectively maintained database. The primary objective was to evaluate whether specific histopathologic factors can reliably predict the likelihood of bilateral disease in low-risk PTC patients after hemithyroidectomy. The secondary objective was to assess the accuracy of preoperative ultrasonography for patients with bilateral disease.

**Results:**

Of the 737 patients in this study, 194 (26.3%) had bilateral disease. The multivariate analysis showed that larger median tumor size (odds ratio [OR] 1.043 per mm; 95 % confidence interval [CI] 1.025–1.062; *P* < 0.001), ipsilateral multifocal disease (MFD) (OR 2.010; 95% CI 1.338–3.020; *P* < 0.001), and venous invasion (OR 1.693; 95% CI 1.058–2.707) had a significant association with bilateral disease. However, in the prediction of clinically significant contralateral disease (≥ 10 mm), median tumor size (OR 1.104 per mm; 95% CI 1.059–1.152; *P* < 0.001) and venous invasion (OR 2.815; 95% CI 1.044–7.589; *P* = 0.041) were significantly correlated, whereas ipsilateral MFD lost its significance. These significant contralateral tumors were identified preoperatively and associated with higher Thyroid Imaging, Reporting and Data System (TIRADS) and/or Bethesda cytology classifications in 94% of cases.

**Conclusion:**

In low-risk PTC patients, larger tumor size, venous invasion, and ipsilateral MFD are significantly associated with disease in the contralateral thyroid lobe.

**Supplementary Information:**

The online version contains supplementary material available at 10.1245/s10434-024-16352-z.

In recent years, there has been a significant shift toward adopting a more conservative approach for the treatment of patients with low-risk papillary thyroid cancer (PTC). The American Thyroid Association (ATA) 2015 guidelines recommend consideration of individual risk factors before provision of a tailored treatment strategy.^1^ Patients categorized as having ATA low-risk PTC typically exhibit favorable prognostic factors and have a low risk of recurrence.

Since 2015, the ATA recommends consideration of hemithyroidectomy (HT) instead of total thyroidectomy with or without radioactive iodine (TT ± RAI) for these patients. To be considered “low risk,” patients must not have any known high-risk factors such as tumor larger than 4 cm, extrathyroidal extension (ETE), tall cell differentiation, and significant lymph node involvement (more than 5 central lymph node metastases and/or lateral lymph node metastasis).^2^ Additional pathologic features important in postoperative risk stratification are lymph invasion and angio-invasion, BRAF mutation status, and tumor multifocality. The prognostic importance of multifocal disease (MFD) for recurrence remains the subject of debate.

Recent studies have highlighted tumor bilaterality as an independent risk factor for tumor recurrence, whereas (unilateral) tumor multifocality does not appear to carry the same significance.^3–6^ These insights could add a critical layer to the decision-making process in (low-risk) thyroid surgery, impacting the choice between HT and TT ± RAI. Notably, bilaterality is not mentioned as one of the possible independent risk factors for recurrence in the ATA risk stratification.^1^ However, it is important to understand the potential significance of bilateral tumors when opting for HT as the definitive surgical management.

This study aimed to investigate the significance of bilateral disease in patients with low-risk PTC as well as the utility of preoperative ultrasonography in identifying significant contralateral tumors. The primary objective was to evaluate whether specific histopathologic factors can reliably predict bilateral disease in low-risk PTC patients after hemithyroidectomy. The secondary objective was to assess the accuracy of preoperative ultrasonography in patients with bilateral disease. We aimed to enhance the postoperative risk stratification process after HT in low-risk PTC guiding the decision for completion thyroidectomy.

## Methods

### Study Design

This retrospective cohort study obtained clinical data from the prospectively maintained thyroid cancer database held within the University of Sydney Endocrine Surgery Unit and Department of Endocrine Surgery at the Royal North Shore Hospital (RNSH) in Sydney, Australia. Ethics approval was obtained from the Northern Sydney Local Health District Ethics Committee (reference 2020/ETH02787). All patients who underwent TT ± RAI for PTC size 0 to 4 cm at the Endocrine Surgery department between 2013 and 2020 were identified from the database. Patients with PTC were included when they met the ATA low-risk criteria,^1^ underwent either total thyroidectomy (TT) or hemithyroidectomy (HT) followed by completion thyroidectomy, and had available information on tumor multifocality and/or bilaterality.

Demographic data, including age at the time of surgery, sex, surgical treatments, clinical characteristics, preoperative ultrasonography, and fine-needle aspiration cytology (FNAC) results, were collected and reviewed. Core data elements from pathology reports, including T stage, N stage, extranodal extension, venous invasion, BRAF mutation status, multifocality, bilaterality, and recurrent disease also were collected and reviewed. The FNAC results were classified according to the 2023 Bethesda System for Reporting Thyroid Cytopathology.^7^ The pathology reports were used to restage according to the TNM eighth edition of the American Joint Committee on Cancer (AJCC) manual for staging.^8^

The study excluded patients younger than 18 years, those with unknown tumor stage (Tx), and those with involvement of lateral lymph nodes at diagnosis (pN1b) or a diagnosis of non-invasive follicular thyroid neoplasm with papillary-like features (NIFTP). Additionally, patients treated with hemithyroidectomy only and those with unavailable follow-up data were excluded from analysis. Finally, patients with specific aggressive PTC subtypes (e.g., tall/columnar/solid and hobnail variants) and those with poorly differentiated, medullary or anaplastic thyroid cancer were excluded.

### End Points and Definitions

Multifocality was defined as two or more foci of thyroid cancer on histopathology. Bilateral disease was defined as the presence of PTC in both the left and right thyroid lobes on histopathology.

The patients were divided into three different groups based on type of multifocality: group A (patients with unifocal disease [UFD]), group B (patients with unilateral MFD), and group C (patients with bilateral disease). The patients with “ipsilateral MFD” were classified as having MFD in the ipsilateral (or index) thyroid lobe with or without the presence of contralateral cancers (bilateral disease). Therefore, this group differed from group B. The ipsilateral thyroid lobe was identified based on preoperative fine-needle aspiration (FNA) cytology site or sites.

To be considered part of primary treatment, completion thyroidectomy, any lymph node dissection (LND), or administration of adjuvant radioactive iodine therapy (RAI) had to be performed within 1 year after initial HT. Reoperation more than 1 year of after initial HT with pathology-proven disease was defined as recurrence.^1^ Data on thyroglobulin levels and/or radioactive iodine single-photon emission computed tomography (SPECT) scans were infrequently available in the utilized database registries. Therefore, recurrence was strictly defined only as pathology-proven recurrence to limit potential information bias. Also, to adjust for the possible delayed treatment of patients with persistent rather than recurrent disease, recurrence was arbitrarily defined as new disease detected more than 1 year after the index treatment. Recurrence was subdivided into locoregional (the thyroid bed, central or lateral cervical nodal levels) or distant (all disease locations other than locoregional recurrence).

Follow-up data were obtained by examining the date of the primary surgery and the most recent date of patient contact. Preoperative ultrasound images were reviewed to evaluate their utility for detecting significant contralateral cancers or lesions with suspicious ultrasonographic features warranting FNAC analysis. “Significant contralateral cancers” were defined as contralateral tumors 10 mm in size or larger on histopathology. Ultrasonography images were compared with histopathology. Tumors matching in size and specific location were assessed and reclassified using the Thyroid Imaging, Reporting and Data System (TIRADS).^9^

### Statistical Analysis

All statistical analyses were performed using SPSS version 28 (SPSS, Inc, Chicago, IL, USA). Baseline characteristics are presented as means ± standard deviations for data with a normal distribution and were compared using Student’s *t* test. Baseline data with a non-normal distribution are presented as medians and were compared using the Mann-Whitney *U* test. Categorical data are presented as percentages and were compared with the chi-square test. Data from continuous variables with a normal distribution from more than two groups were compared using one-way analysis of variance (ANOVA).

Multivariate logistic regression was performed using bilateral disease as the dependent variable. Univariate logistic regression was used for selection of variables and assessment of confounding (if *P* was ≤ 0.05) and effect modification. Significant factors were reported as odds ratios (ORs) with 95 % confidence intervals (CIs). The multivariate logistic regression for bilateral disease accounted for pre-set potential risk factors, with age, sex, median tumor size, N stadium, BRAFV600E mutation status, ipsilateral MFD, and venous invasion included as covariates in the analysis. Extranodal extension and T stage were not included due to their inherent collinearity with median tumor size and lymph node positivity, respectively. In the case of missing data for certain variables, analyses were conducted using only the available data. Missing data were not included in these analyses to ensure that comparisons were based solely on the cases with complete information.

## Results

### Baseline Characteristics

The study included 737 patients treated with TT ± RAI for low-risk PTC according to the inclusion and exclusion criteria, with a median follow-up period of 5.8 months. Baseline patient and treatment characteristics are presented in Table 1. The patients were predominantly female (*n* = 596, 80.9%), and the mean age was 52.2 ± 14.4 years. Slightly more than half of the patients (53.3%, 393/737) had an FNAC Bethesda score of 4 or higher.

**Table 1:** Baseline patient and treatment characteristics according to multifocality and bilaterality

		Multifocal disease (group B+C)				*P* value A–B	*P* value B–C	*P* value A–C
		Total (*n* = 737) *n* (%)	Group A UFD (*n* = 455, 61.7 %) *n* (%)	Group B Unilateral MFD (*n* = 88, 11.9 %) *n* (%)	Group C Bilateral disease (*n* = 194, 26.3 %) *n* (%)			
Mean age (years)		52.23 ± 14.4	51.96 ± 14.8	53.25 ± 13.68	52.41 ± 13.82	0.451	0.637	0.719
Sex	Men	141 (19.1)	92 (20.2)	10 (11.4)	39 (20.1)	0.052	0.073	0.973
	Woman	596 (80.9)	363 (79.8)	78 (88.6)	155 (79.9)			
Median follow-up: months (days)		5.8 (176)	5.5 (169)	8.0 (244)	5.8 (176)	0.279	0.791	0.248
FNA	Beth.1	10 (1.4)	6 (1.3)	1 (1.1)	3 (1.5)	0.061^a^	0.710^a^	**0.002**^a^
	Beth. 2	102 (13.8)	79 (17.4)	7 (8.0)	16 (8.2)			
	Beth. 3	59 (8.0)	36 (7.9)	9 (10.2)	14 (7.2)			
	Beth. 4	78 (10.6)	43 (9.5)	11 (12.5)	24 (12.4)			
	Beth. 5	88 (11.9)	49 (10.8)	16 (18.2)	23 (11.9)			
	Beth. 6	227 (30.8)	128 (28.1)	27 (30.7)	72 (37.1)			
	Not done or N/a	173 (23.5 %)	114 (30.0)	17 (19.3)	42 (21.6)			
Total thyroidectomy	One-stage	662 (89.8)	417 (91.6)	78 (88.6)	167 (86.1)	0.362	0.556	**0.031**
	Two-stage	75 (10.2)	38 (8.4)	10 (11.4)	27 (13.9)			
LND		382 (51.8)	226 (49.7)	50 (56.8)	106 (54.6)	0.220	0.733	0.246
	Prophylactic	358 (93.7)	212 (93.8)	47 (94.0)	99 (93.4)			
	Therapeutic	24 (6.3)	14 (6.2)	3 (6.0)	7 (6.6)			
Radioactive iodine therapy	Yes	242 (32.8)	110 (24.2)	28 (31.8.)	104 (53.6)	0.370	**0.001**	**< 0.001**
	No	291 (39.5)	196 (43.1)	39 (44.3)	56 (28.9)			
	N/a	204 (27.7)	149 (32.7)	21 (23.9)	34 (17.5)			

Significant values are given in bold (*P* < 0.05)

*UFD* unifocal disease, *MFD* multifocal disease, *FNA* fine-needle aspiration, *Beth.* Beth esda, *N/a* data not available, *LND* lymph node dissection

^a^Comparison between the Bethesda 1–3 and 4–6 groups

The patients were predominantly treated with a one-stage TT (*n* = 662, 89.8%) compared with a two-stage TT (*n* = 75, 10.2%). Notably, 51.8 % (382/737) of the patients underwent a LND, 93.7% (358/382) of which were performed with prophylactic intent. Adjuvant RAI was administered to 242 (32.8%) of the patients.

Group A consisted of 455 patients (61.7%) with UFD. The patients with MFD (*n* = 282, 38.3%) were divided into groups B and C based on bilaterality. Group B consisted of 88 (11.9%) patients with unilateral MFD, and group C consisted of 194 (26.3%) patients with bilateral disease. Of the patients in group C, 51 (26.3%) showed ipsilateral MFD. Because 16 (8.2%) of the patients had an unspecified index thyroid lobe (e.g., because FNAC was not performed), they were not categorized into specific groups based on MFD.

Some differences were observed between the patients in groups A and C. The patients in group C presented more often with a preoperative FNA Bethesda result of 4 or higher than the patients in group A (*P* = 0.002). Also, the patients in group C were treated more often with a two-stage thyroidectomy than the patients in group A (*P* = 0.031). Finally, the patients with bilateral disease (group C) were treated more often with adjuvant RAI therapy than those in group A (*P* < 0.001) or group B (*P* = 0.001).

Baseline histopathologic characteristics are presented in Table 2. Of the 737 patients in the study, 415 (56.3%) presented with microcarcinomas. The remaining 43.7% of the patients presented with tumors between 10 and 40 mm in size. The patients with bilateral disease (group C) were observed to present more frequently with a T2 tumor instead of a T1 tumor than the patients with unifocal disease (group A; *P* = 0.025). The patients with bilateral disease were observed to have a median primary tumor size of 12.0 mm. This was statistically larger than in group A (median, 7.0 mm; *P* < 0.001) or group B (median, 8.0 mm; *P* < 0.001). Furthermore, in the patients with bilateral disease, the median tumor size of the largest contralateral cancer was observed to be only 2.3 mm (range, 0.2–20 mm).

**Table 2:** Baseline histopathologic characteristics according to multifocality and bilaterality

		Multifocal disease (group B+C)				*P* value A–B	*P* value B–C	*P* value A–C
		Total (*n* = 737) *n* (%)	Group A UFD (*n* = 455, 61.7 %) *n* (%)	Group B Unilateral MFD (*n* = 88, 11.9 %) *n* (%)	Group C Bilateral disease (*n* = 194, 26.3 %) *n* (%)			
pT stage	T1a	415 (56.3)	282 (62.0)	53 (60.2)	80 (41.2)	0.567^a^	0.059^a^	**0.025**^a^
	T1b	210 (28.5)	111 (24.4)	25 (28.4)	74 (38.1)			
	T2	112 (15.2)	62 (13.6)	10 (11.4)	40 (20.6)			
pN stage	N0	409 (55.5)	250 (54.9)	52 (59.1)	108 (55.7)	0.585^b^	0.898^b^	0.363^b^
	N1a	130 (17.6)	73 (16.0)	18 (20.5)	39 (20.1)			
	Nx	198 (26.9)	132 (29.0)	18 (20.5)	47 (24.2)			
Extranodal extension		10 (1.4)	5 (1.1)	2 (2.3)	3 (1.5)	0.579	0.693	0.887
Ipsilateral MFD	Yes	139 (18.9)	–	88 (100)	51 (26.3)		–	
	No	582 (79.0)	–	0	127 (65.5)			
	Unknown	16 (2.2)	–	0	16 (8.2)			
Median tumor size (mm)	1st tumor	9.0	7.0	8.0	12.0	0.081	**< 0.001**	**< 0.001**
	2nd tumor	2.3	–	1.8	3.0		**< 0.001**	
	3rd tumor	1.5	–	1.0	2.0		0.061	
Median tumor size of largest contralateral tumor (mm)	1st tumor	2.3	–	–	2.3	–	–	–
	2nd tumor	1.5	–	–	1.5	–	–	–
BRAFV600	positive	262 (35.5)	138 (30.3)	35 (39.8)	89 (45.9)	0.081	0.482	0.133
	Negative	117 (15.9)	77 (16.9)	10 (11.4)	30 (15.5)			
	N/a	358 (48.6)	240 (52.8)	43(48.8)	75 (38.7)			
Venous invasion	Yes	98 (13.3)	44 (9.7)	15 (17.0)	39 (20.1)	**0.042**	0.545	**< 0.001**
Surgical margin	R0	719 (97.6 %)	446 (98.0 %)	86 (97.7)	187 (96.4)	0.857	0.869	0.617
	R1	16 (2.2 %)	9 (2.0)	2 (2.3)	5 (2.6)			
	Rx	2 (0.3 %)	0	0	2 (1.0)			
Recurrence		6 (0.8)	3 (0.7)	1 (1.1)	2 (1.0)	0.632	0.936	0.620

Significant values are given in bold (*P* < 0.05)

*UFD* unifocal disease, *MFD* multifocal disease, *Nx* lymph node status cannot be assessed, *N/a* lymph node data not available

^a^Comparison between T1 (T1a and T1b) and T2 tumors

^b^Comparison between N0 and N1a patients

At presentation, 130 (17.6%) patients presented with lymph node metastasis to central compartment lymph nodes. No statistical differences were found between groups A, B, and C regarding this variable. Venous invasion was observed to be present more often in the patients with MFD than in those with UFD in both unilateral MFD (*P* = 0.042) and bilateral (*P* < 0.001) disease.

### Logistic Regression Analysis of Possible Predictors for Bilateral Disease

The results of the uni- and multivariate logistic regression analyses for potential predictors of bilateral disease are presented in Table 3. The univariate analysis demonstrated statistically significant associations between bilateral disease and median tumor size (*P* < 0.001), ipsilateral MFD (*P* < 0.001), and venous invasion (*P* = 0.001). Specifically, larger median tumor size was associated with an increased OR of 1.044 per mm (95% CI 1.027–1.061), whereas the presence of ipsilateral MFD had an increased OR of 2.076 (95% CI 1.396–3.088), and the presence of venous invasion yielded an OR of 2.064 (95% CI, 1.325–3.215). In the multivariate analysis, median tumor size and ipsilateral MFD remained significantly associated with bilateral disease, with slightly adjusted ORs of 1.043 per mm (95 % CI 1.025–1.062; *P* < 0.001) and 2.010 (95 % CI 1.338–3.020; *P* < 0.001), respectively. Venous invasion also retained its statistical significance in the multivariate model, with an adjusted OR of 1.693 (95 % CI 1.058–2.707; *P* = 0.028). Other factors, including male sex, age of 55 years or older, N+ stadium (N1a), and positive BRAFV600 mutation status, did not show statistically significant associations with bilateral disease.

**Table 3:** Logistic regression odds model for bilateral disease

Characteristics	Univariate		Multivariate	
	OR (95 % CI)	*P* value	OR (95 % CI)	*P* value
Male sex	1.088 (0.721–1.642)	0.689		
Age ≥ 55 years	0.918 (0.661–1.276)	0.612		
Median tumor size	1.044 (1.027–1.061)	**< 0.001**	1.043 (1.025–1.062)	**< 0.001**
N+ stadium (N1a)^a^	1.194 (0.773–1.845)	0.423		
BRAFV600+	1.280 (0.827–1.981)	0.268		
Ipsilateral MFD^b^	2.076 (1.396–3.088)	**< 0.001**	2.010 (1.338–3.020)	**< 0.001**
Venous invasion+	2.064 (1.325–3.215)	**0.001**	1.693 (1.058–2.707)	**0.028**

Significant values are given in bold (*P* < 0.05)

*OR* odds ratio, *CI* confidence interval, *MFD* multifocal disease

^a^Comparison between patients with N0 and those with N1a

^b^Patients with multifocal disease in the ipsilateral or “index” thyroid lobe

Logistic regression analysis for potential predictors of bilateral disease of tumors 10 mm or larger is presented in Table 4. In the multivariate analysis, the presence of contralateral tumors 10 mm or larger was significantly correlated with median tumor size (OR 1.104; 95 % CI 1.059–1.152; *P* < 0.001) and venous invasion (OR 2.815; 95 % CI 1.044–7.589; *P* = 0.041). Ipsilateral MFD did not show a significant correlation with the presence of contralateral tumors 10 mm or larger (*P* = 0.654), whereas it was shown to be an independent predictor for bilateral disease of any size (*P* < 0.001).

**Table 4:** Logistic regression odds model for bilateral disease size ≥10 mm

Characteristics	Univariate		Multivariate	
	OR (95% CI)	*P* value	OR (95% CI)	*P* value
Male sex	1.131 (0.370–3.461)	0.829		
Age ≥ 55 years	1.041 (0.418–2.591)	0.932		
Median tumor size	1.107 (1.063–1.152)	**< 0.001**	1.104 (1.059–1.152)	**< 0.001**
N+ stadium (N1a)^a^	1.778 (0.585–5.403)	0.310		
BRAFV600+	0.811 (0.302–2.176)	0.677		
Ipsilateral MFD^b^	1.297 (0.416–4.040)	0.654		
Venous invasion +	4.019 (1.542–10.473)	**0.004**	2.815 (1.044–7.589)	**0.041**

Significant values are given in bold (*P* < 0.05)

*OR* odds ratio, *CI* confidence interval, *MFD* multifocal disease

^a^Comparison between patients with N0 and those with N1a

^b^Patients with multifocal disease in the ipsilateral or “index” thyroid lobe

### Recurrence

Of the 737 patients, 6 had structural recurrent disease. Four of these six patients had recurrence in one or more lateral lymph nodes. One patient presented with metastatic poorly differentiated thyroid carcinoma arising from PTC 81 months after initial surgery, which showed low-risk PTC on initial histopathology. The remaining patient presented with recurrence in the thyroid bed. No statistical differences were observed between groups A, B, and C.

The results of the logistic regression analysis for potential predictors of recurrence are presented in Table 5. In the univariate logistic regression analysis, median tumor size was found to be a significant predictor of recurrence (OR 1.093; 95% CI 1.022–1.170; *P* = 0.010). Positive nodal status (N1a) approached statistical significance as a predictive factor (OR 9.638; 95% CI 0.994–93.467; *P* = 0.051). Other factors, including multifocality and bilaterality, did not show significant associations with recurrence. However, the event rate was too low to draw meaningful conclusions.

**Table 5:** Logistic regression odds model for recurrence

Characteristics	Univariate		Multivariate	
	OR (95% CI)	*P* value	OR (95% CI)	*P* value
Male sex	0.844 (0.098–7.284)	0.878	–	
Age ≥55 years	1.156 (0.232–5.767)	0.859	–	
Median tumor size	1.093 (1.022–1.170)	**0.010**	–	
N+ stadium (N1a)^a^	9.638 (0.994–93.467)	0.051	–	
BRAFV600 +	2.310 (0.256–20.860)	0.456	–	
Multifocality	1.620 (0.325–8.083)	0.556	–	
Unilateral MFD^b^	1.480 (0.171–12.820)	0.722	–	
Bilateral disease	1.404 (0.255–7.725)	0.697	–	

Significant value given in bold (*P* < 0.05)

*OR* odds ratio, *CI* confidence interval, *MFD* multifocal disease

^a^Comparison between patients with N0 and those with N1a

^b^Patients with multifocal disease in the ipsilateral or “index” thyroid lobe

### Preoperative Ultrasonography of Patients with Contralateral PTC 10 mm or Larger

Of the 194 patients with bilateral disease, 175 (90.2%) had contralateral tumors smaller than 10 mm. Of the 19 (9.8%) remaining patients, 17 had ultrasound images available for analysis. These were reviewed and are presented in Table 6. The median tumor diameter on histology was 20 mm. Regarding the TIRADS classification, 36.8% of the patients were classified as TIRADS 4, whereas 21.1% were classified as TIRADS 5. It was impossible to reclassify the TIRADS score of eight patients (42.1%) because raw image data were not available. None of the patients had a TIRADS classification of 3 or lower. A high percentage (94.1%) of the patients showed a suspicious nodule on preoperative ultrasound. Additionally, 78.9% of the patients had preoperative FNAC performed or indicated, whereas in only one case (5.8%), FNAC was not indicated.

**Table 6:** Preoperative ultrasound characteristics of patients with contralateral lobe PTC size > 10 mm

	*n* (%)
Total no. of patients	19
Total ultrasound images available	17 (89.5)
Median tumor diameter on histology (mm)	20.00
TIRADS	
1 or 2	0
3	0
4	7 (36.8)
5	4(21.1)
N/a^a^	8 (42.1)
Suspicious contralateral nodule on preoperative US^b^	16 out of 17 (94.1)^c^
Contralateral nodule preop FNA performed or indicated	
Yes	15 (78.9)
No	1 (5.3)
N/a	3 (15.8)

*TIRADS* thyroid imaging, reporting and data system; *N/a* data not available, *US* ultrasound, *FNA* fine-needle aspiration

^a^Not available due to insufficient (quality or quantity) of images for accurate assessment of the TIRADS score

^b^US concordant in size and site compared with histology

^c^The remaining patient had a nodule on the concordant site, but histopathology size was greater than on US (20 and 10 mm, respectively; FNA would have been indicated based on images in this case).

Only two of the six patients who experienced recurrence, had preoperative ultrasound available for analysis. Both patients showed bilateral disease on pathology. In the first case, the ultrasound showed a multinodular goitre of both thyroid lobes. The primary tumor was classified as a Bethesda 5 after FNA and treated with a one-stage TT. The contralateral nodule, not detailed in the ultrasound report, measured 5 mm on pathology. In the second case, the ultrasound identified only one nodule, analyzed by FNA, and noted a heterogeneous echotexture with bilateral areas of reduced echotexture suggestive of lymphocytic infiltration. The bilateral tumor measured 1.5 mm on pathology, and the patient also underwent a one-stage TT.

## Discussion

This retrospective cohort study aimed to investigate the predictive factors for bilateral disease in patients with low-risk PTC, as well as the role of preoperative ultrasonography in detecting clinically significant contralateral tumors. The multivariate analysis showed that bilateral disease was significantly associated with a larger median tumor size (OR 1.043 per mm; 95% CI 1.025–1.062; *P* < 0.001), ipsilateral MFD (OR 2.010; 95% CI 1.338–3.020; *P* < 0.001), and venous invasion (OR 1.693; 95 % CI 1.058–2.707). This is in keeping with the current available literature reporting on predictive histopathologic factors for bilateral disease, which is limited and primarily focused on patients with papillary thyroid microcarcinomas (PTMCs).^10–14^ The multivariate analysis for the presence of bilateral disease 10 mm or larger showed a significant correlation with larger median tumor size (OR 1.104 per mm; 95% CI 1.059–1.152; *P* < 0.001)) and venous invasion (OR 2.815; 95% CI 1.044–7.589; *P* = 0.041), whereas ipsilateral MFD lost its significance in this cohort, suggesting that bilateral disease predicted by ipsilateral MFD may not be of clinical significance, especially when considered in the context with high-resolution preoperative ultrasonography.

In our recent paper published in *JNCI*^3^, we reported on treatment outcomes for Dutch low-risk PTC patients: We observed that bilateral disease was an independent risk factor for recurrence.^3^ This is supported by several other recent papers.^4–6,15^ The results reported in this paper suggest that larger tumor size, venous invasion, and ipsilateral MFD are independent predictors for bilateral disease and should therefore be taken into account when opting a HT instead of TT or completion thyroidectomy. However, further granular detail regarding the nature of the bilateral disease is important because a 1-mm contralateral deposit PTC likely differs prognostically from a 15-mm contralateral tumor. The median size of contralateral tumors in our patient cohort was 2.3 mm.

The clinical significance of these small tumors is debatable, particularly during follow-up evaluation after HT for patients with low-risk PTC. However, bilateral disease remains reported as an independent risk factor for recurrence.^3–6^ We defined clinically significant contralateral tumors as those measuring 10 mm or larger because these would warrant consideration for FNA during follow-up evaluation. The significance of smaller contralateral tumors (< 10 mm) is less clear, and we could not determine whether a larger median contralateral tumor size correlates with a higher risk of recurrence. For these clinically significant contralateral tumors, ipsilateral MFD was not found to be predictive in the multivariate analysis.

Interestingly, unilateral MFD was not found to be an independent risk factor for recurrence in our Dutch cohort either. This raises important considerations regarding the necessity of completion thyroidectomy in such cases. Our study suggests that unilateral MFD alone may not warrant completion thyroidectomy unless accompanied by other significant factors such as large primary tumor or venous invasion. Furthermore, the presence of suspicious nodules in the contralateral lobe on ultrasound may serve as an additional indicator for intervention. We reviewed and evaluated ultrasonography images of patients with contralateral tumors measuring 10 mm or larger on histopathology. Our analysis showed that the malignant nodules were identified preoperatively and associated with high TIRADS, Bethesda cytology classifications, or both in 94.1% of cases. Consequently, the likelihood of missing significant bilateral tumors when opting for HT appears negligible when the results of high-resolution preoperative ultrasonography are also considered. It is important to note that thyroid nodules are common in the healthy population, with prevalence ranging from 4 to 7% by palpation and reaching 20–76 % on neck ultrasonography, which may lead to overestimation of incidentalomas. Despite the high detection rates for nodules, the actual incidence of cancer remains relatively low.^16,17^

In our Dutch cohort, 22 patients showed disease recurrence, with 20 (2.8%) located in the thyroid bed.^3^ Conversely, in the Australian cohort, the disease recurrence for the majority of the patients appeared within the lateral lymph nodes, likely due to the routine performance of central LND. The recurrence rates in both cohorts seemed low and in line with current literature.^18–22^ However, it must be noted that the true recurrence rates may have been underestimated in a de-escalated setting given that all the patients in both the Dutch and Australian cohorts had been treated with TT ± RAI. Except for that, the Dutch and Australian cohort seemed reasonably comparable as both studies had similar inclusion criteria as well as similar workup and treatment strategies (Table S1), regardless of time span differences. Also, the rates of multifocality and bilaterality were similar between the cohorts. The main differences between the two cohorts lay in the different inclusion time periods and the standard prophylactic central LND, which was routinely performed in the Australian cohort. Notably, in the *JNCI* Dutch cohort, only 5 (0.6%) of the 791 patients had received a LND, and 80.4% of the patients were staged as pNx, which was considerably higher in our Australian cohort.^3^ In this current study, positive nodal status approached significance as a predictive factor for disease recurrence (OR 9.638; 95% CI 0.994–93.467; *P* = 0.051), and the significance of this analysis was likely limited by the low recurrence event rate in this cohort.

Overall, these findings raise questions about the differences in tumor biology between types of multifocality and bilateral disease. Further research is needed to understand the underlying mechanism and clinical implications of bilateral disease in PTC. Ipsilateral MFD could be associated with bilateral disease due to the presence of germline variant-mediated field change in the thyroid parenchyma, predisposing to malignant transformation. Furthermore, some benign thyroid diseases (nontoxic goitre, simple goitre, hyperthyroidism, thyroid adenoma) are associated with an increased incidence of clinically insignificant PTMC,^23^ possibly explaining the observed difference between overall multivariate analysis and multivariate analysis predicting bilateral tumors 10 mm in size or larger. The relative increase in recurrence risk conferred by clinically significant bilateral tumors requires further evaluation.

Several limitations of this study must be considered. Inherent to the retrospective study design, missing data in pathology reports account for a bias in the estimation of parameters. Importantly, comprehensive and statistical evaluation of the value of preoperative ultrasonography in predicting bilateral disease was limited by the lack of raw image data and inconsistent reporting practices by radiologists. However, the database at the RNSH has been prospectively maintained with monthly surgeon-led auditing of the accuracy of the data. In addition, the retrospective nature of the study gives rise to the possibility of a selection bias in patients offered TT ± RAI or completion thyroidectomy.

## Conclusion

In patients with low-risk PTC, larger tumor size, venous invasion, and ipsilateral MFD are associated with the occurrence of malignant disease in the contralateral lobe.

## Supplementary Information

Below is the link to the electronic supplementary material.

Supplementary file1 (DOCX 14 KB)

## Data Availability

The data underlying this article will be shared on reasonable request to the corresponding author.
