# Supplementary material for: Predictors of Bilateral Disease in Low-Risk Papillary Thyroid Cancer: Histopathologic Insights and Preoperative Ultrasonography
Source: Ann Surg Oncol. 2025 Jan 14;32(4):2335–43. doi: 10.1245/s10434-024-16352-z (PMC11882697; doi:10.1245/s10434-024-16352-z)
Supplement: Supplementary file 1 — Supplementary file1 (DOCX 14 KB) [file 10434_2024_16352_MOESM1_ESM.docx]

| **Supplementary Table 1. Comparison cohort between Dutch and Australian population** | | |
| --- | --- | --- |
| Characteristics | Total  *Dutch cohort*  (n = 791) | Total  Sydney Cohort (n=737) |
| Inclusion period Age, mean (SD) | **2005-2015**  46.36 (13.55) | **2013-2020**  52.22 (14.4) |
| Sex, No. (%) |  |  |
| Men | 135 (17.1) | 141 (19.1) |
| Women | 656 (82.9) | 596 (80.9) |
| Follow-up median time, y | **8.0 years** | **5.8 months** |
| pT stage, No. (%) |  |  |
| T1a | 176 (22.3) | 415 (56.3) |
| T1b | 281 (35.5) | 210 (28.5) |
| T2 | 334 (42.2) | 112 (15.2) |
| pN stage, No. (%) |  |  |
| Nx | 636 (80.4) | 198 (26.9) |
| N0 N1a | 155 (19.6)  0 | 409 (55.5)  130 (17.6) |
| Radioactive iodine therapy, No. (%) | 663 (83.8) | 242 (32.8) |
| Neck dissection, No. (%) | 5 (0.6) | 382 (51.8) |
| Unifocal disease (UFD)  Unilateral MFD  Bilateral disease Recurrence, No. (%) | 460 (58.2)  103 (13.0)  228 (28.8)  22 (2.8) | 455 (61.7)  88 (11.9)  194 (26.3)  6 (0.8) |
